# Evaluation of residual bone mass of the mandibular third molar and the risk of mandibular fracture

**DOI:** 10.4317/medoral.26520

**Published:** 2024-05-25

**Authors:** Jingying Mu, Chunfeng Wu, Linjun Ouyang, Yongzhen Yang, Yuna Wu, Bin Jin

**Affiliations:** 1 Department of Stomatology, Baicheng Medical College, Jilin Baicheng, China; 2 Department of Stomatology, Yanbian University Hospital, Jilin Yanji, China; 3 The 32183 Troops Hospital of PLA, China; 4 Department of Cardiology, Yanbian University Hospital, Yanji, Jilin Province, China; 5 Department of Post-Doctoral Research Center, Yanbian University Hospital, Yanji, Jilin Province, China

## Abstract

**Background:**

A quantification of the residual bone mass of the mandible (B/A) was utilized in this study to examine the correlation between mandibular fracture and residual bone mass. To improve the clinical utilization rate and reduce the incidence of iatrogenic mandibular fractures, the B/A ratio calculation should be simplified.

**Material and Methods:**

Data were collected from the Yanbian University Hospital on 175 cases of mandibular fracture with third molar (M3), 67 normal cases without fractures and 20 cases of impacted teeth extraction. Twenty cases of iatrogenic mandibular fracture were collected, and the case records and panoramic radiographs of the patients were recorded.

**Results:**

The average B/A ratio of mandibular angle fracture group was 0.61±0.10.The value of B/A was found to be statistically significant in terms of whether M3 emerged from alveolar bone (*P* = 0.001), location (horizontal *P* < 0.001, vertical *P* < 0.001), the degree of impaction (*P* < 0.001), the number of roots (*P* < 0.001), the difference in impaction (*P* < 0.001), and the fracture type (*P* = 0.002). The average B/A ratio of normal group was 0.62±0.10. In the statistical results of the B/A value of normal patients, M3 involving alveolar bone (*P* < 0.001), position classification (*P* < 0.05), degree of impaction (*P* < 0.001) and presence or absence of a root (*P* < 0.05) were statistically significant. The average B/A ratio of iatrogenic mandibular angle fracture group was 0.28±0.08. The average B/A ratio of the extraction group for impacted teeth was 0.62 ± 0.09.

**Conclusions:**

There is a high risk of mandibular angle fracture when the (B/A) value of the residual bone height (B) in the mandibular M3 area compared to the mandibular bone height (A) in the M3 area is less than 0.4.

** Key words:**Residual bone height (B/A), mandibular angle fracture, tooth extraction risk, panoramic radiographs, third molar, orthopedics.

## Introduction

The weak parts of the mandible include the mandibular angle, the condyle, and the mental foramen. The mandibular angle, as the transition zone between the toothed area and the edentulous area, is usually related to impacted teeth (1). Some academics have proposed a connection between the position of the mandibular M3 and trauma-induced mandibular angle fracture (2). The complications of mandibular third molar (M3) extraction include tissue edema, hemorrhage, infection, dry socket, nerve injury, temporomandibular joint symptoms, and so on (3-5). A meta-analysis revealed that the incidence of iatrogenic mandibular fracture ranged from 0.0034% to 0.0075%;Seventy-five percent of these instances were associated with the extraction of the mandibular M3, while the postoperative fracture of the mandibular M3 accounted for 0.0046% to 0.0075% (4).A mandibular fracture is one of the rare complications of tooth extraction. The incidence of fracture during surgery is 0.0033% to 0.0036% (1,6),and the incidence of fracture after surgery is 0.0046% to 0.0049% (7,8). Although the incidence of iatrogenic mandibular fractures is relatively low, it is considered a serious complication that should draw the attention of oral health professionals. However, there is limited research on the remaining bone volume in the mandibular angle region, and clinicians often rely on clinical experience to plan procedures like the extraction of impacted teeth, aiming to prevent serious complications. Given the advantages of panoramic X-rays over computed tomography in terms of radiation exposure and cost, we chose readily available panoramic slices for data measurement. This study measured the distribution of the ratio of remaining bone height (B) to mandibular bone height (A) in the mandibular third molar region among four groups (traumatic fracture group 174 cases, normal group 67 cases, iatrogenic fracture group 20 cases, impacted tooth extraction group 20 cases) in the population. The objective of this study is to explore the relationship between residual bone mass in the mandibular angle area and the incidence of mandibular fractures. By quantifying this correlation, the study aims to provide empirical support for maxillofacial surgeons, thereby aiding in the reduction of severe complications associated with mandibular fractures.

## Material and Methods

- Research participants and methods

An analysis was conducted on the cases and panoramic radiographs of patients admitted to the Yanbian University Hospital between 2012 and 2023. Out of these, 175 cases were identified as having mandibular fractures, and 67 cases were categorized as non-fracture patients. In addition, 20 cases of iatrogenic mandibular fractures were also compiled from the medical literature. The sex, age, location of M3, and location of the mandibular fracture was recorded, and the ratio of the residual bone mass of the mandible (B/A) of M3 was analyzed. The collected data were statistically analyzed using SPSS 26.0 software. The independent sample t-test or one-way ANOVA were used for comparison between measurement data groups. The chi-squared test was used for comparison between counting data groups. The difference was considered statistically significant at *P* < 0.05. Exclusion criteria: cases with edentulous jaws, incomplete medical records, and inadequate diagnosis.

The highest point of mandibular M3 passes through the alveolar bone during an eruption. The horizontal position (Class I, Class II, and Class III) and vertical position (Class A, Class B, and Class C) of M3 are defined according to the Pell and Gregory (P&G) classification. It can be divided into shallow obstruction (IA, IB, IIA, IIB, or IIIA) and deep obstruction (IC, IIC, IIIB, or IIIC) based on the degree of obstruction. As stated by Ma’aita and Alwrikat (9), the angle of M3 is defined as the measurement between the longitudinal axis of the tooth and the occlusal plane. This angle can be further divided into distoangular (more than 100°), vertical (81 to 100°), mesioangular (between 21 and 80°), and horizontal (less than 20°).

- Measurement method of B/A value

**Figure 1:**
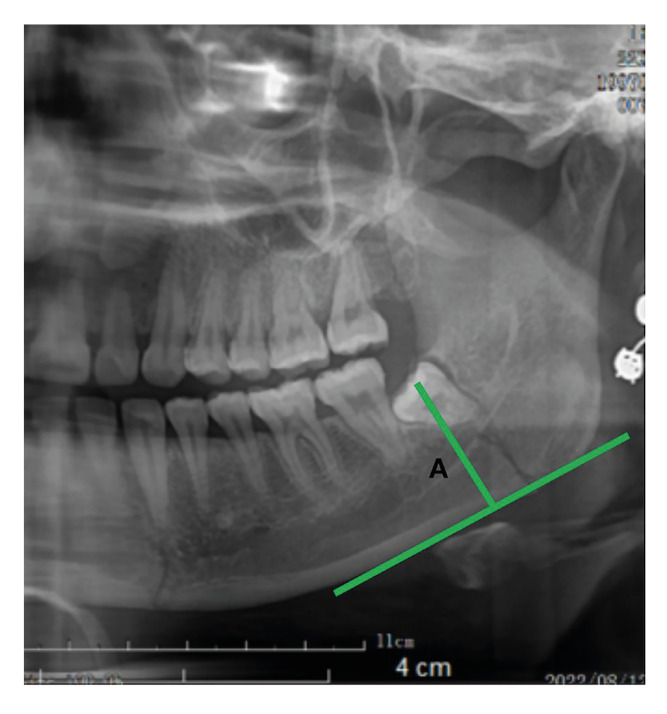
Vertical distance from the midpoint of the mesiodistal alveolar bone of the M3 crown to the inferior border of the mandible(A).

**Figure 2:**
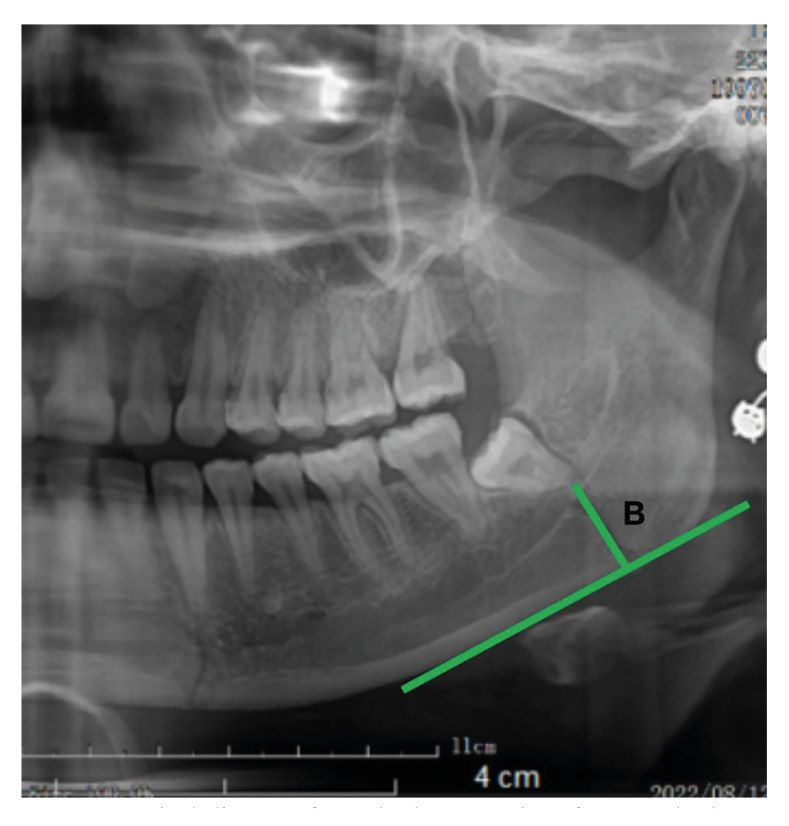
Vertical distance from the lowest point of M3 to the lower edge of the mandible (B).

According to Tateyuki (10), the method for determining mandibular height is as follows:

1. Measurement method of distance A: distance A refers to the vertical distance from the midpoint of the mesiodistal alveolar bone of the M3 crown to the inferior border of the mandible (Fig. 1).

2. Measurement method of distance B: vertical distance from the lowest point of M3 to the lower edge of the mandible (B) (Fig. 2).

The A and B values of each case were measured, and the B/A ratio was calculated.

## Results

- Statistical results of B/A

Fracture Group (Table 1): There were 175 cases in the fracture group, with a male-to-female ratio of 3.86:1. The value of B/A was found to be statistically significant in terms of whether M3 emerged from alveolar bone (*P* = 0.001), location (horizontal *P* < 0.001, vertical *P* < 0.001), the degree of impaction (*P* < 0.001), the number of roots (*P* < 0.001), the difference in impaction (*P* < 0.001), and the fracture type (*P* = 0.002).

The highest proportion of patients in male fracture group is 0.61-0.7 (39.6%), and that in female fracture group is 0.5-0.6 (38.9%).

The B/A value of M3 alveolar bone without eruption was relatively low.The B/A value of the root number group was higher than that of the multi-root group, single root group, and non-root group, and it decreased accordingly.

The B/A ratio of impacted teeth with unerupted, edentulous, or single roots was hypothesized to be comparatively low. The B/A value of impacted teeth without eruption, rootless, or single root was relatively small.

The ratio of B/A was statistically significant in the classification of the position of the M3 in alveolar bone. However, the ratio of the deeply impacted M3 was relatively small, indicating that the deeply impacted M3 is a risk factor for iatrogenic mandibular angle fracture. This result may be related to the large area of alveolar bone occupied by deep-impacted M3 in the mandibular angle area.

Normal non-fracture group: The normal group comprised 67 cases with third molars, spanning an age range of 7 to 78 years and exhibiting an average age of 32 years (range: 23-48). The average B/A for men was 0.63, and that for women was 0.62; therefore, the average B/A for men is slightly larger than that of women. The B/A value of the group under 14 years old was statistically significant compared with other groups (*P* < 0.05). This may be due to the fact that the root of the M3 of patients under 14 years old has not yet developed. In the statistical results of the B/A value of normal patients, M3 involving alveolar bone (*P* < 0.001), position classification (*P* < 0.05), degree of impaction (*P* < 0.001) and presence or absence of a root (*P* < 0.05) were statistically significant. (Table 2)

B/A value of an iatrogenic mandibular fracture: A literature review was conducted to identify 20 cases of iatrogenic mandibular fractures (17 cases of mandibular angle fractures; case E involved a mandibular ramus fracture; case H involved a body fracture; case T was a fracture caused by tooth extraction after mandibular angle osteotomy). The male-to-female ratio was 1:3, with an average age of 37 years for the study participants. There were 11 cases that occurred during surgery, and 9 cases were fractured within 4 weeks after the surgery due to occlusion and other factors.

There were 17 cases of mandibular angle fracture caused by the M3 (not including 3*cases), and the average B/A ratio was 0.28±0.08. The B/A value (0.22-0.27) of the depth (C,III) is small. When considering individual cases, it is hypothesized that mandibular angle fractures are more likely to occur in cases where the depth of the M3 is horizontal or vertical. The average B/A of males (0.31±0.06) was slightly larger than that of females (0.26±0.08). There were 8 cases of fracture during surgery and 9 cases of fracture after surgery, with an average time of 19 days (10-28 days) after surgery. All men suffered from postoperative fractures, with an average time of 22 days (15-28 days). According to the statistical data, with age, the residual bone mass tends to decrease (Table 3).

The B/A value of the extraction group for impacted teeth: The group comprised 20 cases, spanning an age range of 18 to 53 years and exhibiting an average age of 30 years. The B/A average of 0.62±0.09.

Comparison of B/A values among the normal non-fracture group, traumatic fracture group, iatrogenic fracture group, and impacted teeth extraction group: The comparisons of B/A values between the iatrogenic fracture group and the normal group (*P* < 0.05), traumatic fracture and iatrogenic fracture (*P* < 0.05), and the four groups (*P* < 0.001) were all statistically significant. The significantly reduced residual bone mass observed in cases of iatrogenic mandibular angle fracture implies that maxillofacial surgeons should assess the residual bone mass prior to tooth extraction.

In conclusion, the B/A values are arranged as follows: non-fracture group (0.62±0.10),traumatic fracture group(0.61±0.10) ,iatrogenic mandibular angle fracture group (0.28±0.08), impacted teeth extraction group(0.62±0.09). In the traumatic fracture group, the average value of the chin fracture group was the highest (0.65), followed by the condyle fracture group at 0.60 (0.52-0.65), and the mandibular angle fracture group at 0.58 (0.53-0.68) (Table 4).

## Discussion

A significant correlation has been observed between the traumatic mandibular angle fracture and the position of the M3 (11). Their correlation was also established in another study that we conducted. Postoperative fracture risk is associated with the classifications of II or III impaction and B or C impaction (12,13). According to our statistical data, the B/A of deep impaction is relatively small; The incidence rate of iatrogenic mandibular fracture (postoperative) peaked at 67.8% in the second and third weeks after surgery, and affected patients were predominantly older than 25 years (14). Certain researchers identified 28 postoperative fractures in a sample of 611,000 tooth extractions by administering questionnaires to maxillofacial surgeons; Their conclusion suggests that male patients aged over 25 should adhere to a soft food diet for four weeks following tooth extraction (7). Xu *et al*. reached the same conclusion (15). Some researchers have concluded that this is probably due to the fact that men have more chewing power than women (16). Male patients in our iatrogenic fracture group experienced only postoperative fractures, and the average fracture time was 22 days (15-28 days) after the surgery. Recent research indicates that the risk of postoperative fracture is elevated in men aged 35 and older (12). Combined with our data, the risk of iatrogenic fracture increases with age.

A female patient who underwent extraction of the left mandibular third molar 20 days prior presented with a mandibular angle fracture caused by peanut consumption. Correa *et al*. documented an identical case of fracture 15 to 20 days after tooth extraction (17). The difference lies in the presence of osteoporosis and the advanced age of our female patient.When osteoporotic women bite with normal force, their bone resistance is diminished, which increases their susceptibility to iatrogenic fractures (18).

The United States Preventive Services Task Force (USPSTF) recommends osteoporosis screening for women over the age of 65 because of the increased risk of fractures among women over the age of 60; Fracture risk factors include a personal or family history of osteoporosis, a history of fragile fractures after the age of 50, premature menopause, systemic factors (e.g., hyperparathyroidism, hyperthyroidism, malnutrition, rheumatoid arthritis, diabetes, chronic liver or kidney disease), or lifestyle factors (e.g., smoking, eating disorders, alcoholism) (19). Obviously, inflammation of the third molars, cysts, widening of periodontal ligament space, and other pathological factors in the mandibular angle area are also included (6). In the case of a larger cyst of the M3, when the B/A value is low, the treatment plan is adjusted to some extent, such as delayed operation, windowing decompression, cyst reduction, and then surgical treatment to prevent severe complications such as fracture.

In a study involving 189 patients, it was confirmed that there is no difference between the left and right sides of the fracture (20). However, the results of Guillaumet *et al*. indicate that intraoperative mandibular fractures are more common in the left mandible, while postoperative fractures are more prevalent on the right side (12). Statistical analysis revealed that there is a higher incidence of traumatic mandibular angle fractures on the left side compared to the right side.

Some studies indicated that iatrogenic mandibular fractures may also occur even when experienced surgeons are involved (3), hence it is necessary to evaluate the surgical risk through clinical examination and imaging examination before tooth extraction. We maintain that the implementation of B/A is relatively straightforward, and after experienced surgeons have acquired mastery, they can make rough risk assessments without precise measurements. This increases the probability of its adoption in clinical settings.

Research analyzing six cases has underscored the necessity of further investigation into iatrogenic mandibular angle fractures, particularly concerning the B/A ratio, which has consistently been below 0.3.The current lack of a unified standard for measuring the B/A ratio underscores the urgency for establishing reliable, standardized measurement criteria. Our adoption of an alternative measurement method, focusing on the midpoint of the line connecting the mesial and distal alveolar bones of the M3 crown, underscores the necessity for flexibility in clinical practice. However, it also suggests that our measurements may be larger than those derived from traditional center of gravity methods. Future research should aim to validate the B/A ratio through a larger dataset and explore the implications of different measurement techniques on clinical outcomes. Establishing a standardized approach will not only enhance the reliability of research findings but also improve clinical decision-making in the management of mandibular angle fractures. Predicting the difficulty of the extraction of impacted teeth is the key to preoperative design. Operators must utilize existing literature, advanced equipment, and personal experience to understand the risk of iatrogenic mandibular fracture and prevent the occurrence of this complication (21).

Analysis of iatrogenic mandibular fracture cases allows us to infer the following: Vertical depth impactions, covering a larger mandibular area, yield a relatively low B/A ratio, indicating that regions with a smaller B value (weaker areas) are more susceptible to mandibular angle fractures. Similarly, horizontal depth impactions, which affect the weaker mandibular ramus, also increase the risk of iatrogenic mandibular fractures.

In Case E (21), the fracture within the mandibular ramus raises the question of whether the B/A ratio's application should be expanded beyond the mandibular angle. This case also suggests reconsidering the definition of the B value as the shortest distance from the mandibular M3 to the mandible's edge, potentially offering a more accurate metric for fracture risk assessment. Meanwhile, Case H (22), involving a fracture following the extraction of the left mandibular second molar, indicates that areas with less bone surrounding the second molar might require simultaneous preoperative risk assessment and B/A ratio evaluation with extraction. These findings suggest that the B value should be adapTable based on the specific conditions of the mandible.

Case T (15), a 20-year-old female patient requiring plastic surgery, had an intraoperative fracture during the extraction of the mandibular M3 after osteotomy in the mandibular angle region. Moreover, an analysis of B/A indicated a preoperative value of 0.42, while the mean value for females was 0.62 ± 0.10, falling below the normal range. In response to the aesthetic preferences of the patients, an osteotomy of the mandibular angle was executed during the surgery, contributing to a further reduction in the B value. The B/A value after osteotomy was determined to be 0.28, while the range of the B/A value of an iatrogenic mandibular fracture was 0.12-0.39. Hence, in mandibular plastic surgery, if extraction of the M3 is required, it is advisable to perform the tooth extraction before proceeding with the bone-cutting operation. When impacted teeth necessitate extraction during osteotomy, evidence suggests that the residual bone mass of the mandible is lower than normal when the post-osteotomy B/A is less than 0.4. The consideration of incorporating a regimen of light occlusion after 4 weeks, in addition to the physician's guidance, to prevent the occurrence of delayed mandibular fractures represents a potentially novel perspective for plastic surgeons.

In conclusion, it is reasonable to infer that the risk factors for patients with iatrogenic fracture of mandibular angle may include: small B/A (when it is less than 0.4); patients under the age of 14 who have no M3 root formation (requiring orthodontic treatment and extraction, and so on.); and older female patients with vertical or horizontal impaction of M3 and concurrent osteoporosis. It is suggested that oral surgeons may be able to anticipate this risk factor before extracting mandibular M3, including through the development of a preoperative surgical plan. A gentle approach to tooth extraction is recommended during the operation to mitigate the potential risk. Particular attention should be paid to preserving the bone in the mandibular angle, especially the cortical bone of the external oblique ridge (1). Patients should be advised to consume soft foods within 4 weeks after the operation. Furthermore, the application of a mini titanium plate can effectively prevent the occurrence of mandibular fracture (23) and avoid serious complications (fractures) in patients with high fracture risk.

## Conclusions

1. B/A ratio below 0.4 significantly increases the risk of mandibular angle fractures.

2. The B value should not be confined to the M3 area, and considerations of residual bone mass may be extend beyond the mandibular angle, applicable to aesthetic dental surgery as well.

3. Vertical or horizontal impaction of depth serves as a risk factor for iatrogenic mandibular fracture.

4. Panoramic radiographs represent an efficient method for predicting this risk.

## Figures and Tables

**Table 1:** B/A value of fracture patients.

		Number of cases	<0.5	0.5-0.6	0.61-0.7	>0.7	χ2	P
Gender, n (%)	Male	139	20(14.4)	41(29.5)	55(39.6)	23(16.5)	2.289	0.515
	Female	36	6(16.7)	14(38.9)	13(36.1)	3(8.3)		
Age, n (%)	<14	3	1(33.3)	2(66.7)	0(0.0)	0(0.0)	12.694	0.383
	14-24	51	10(19.6)	21(41.2)	14(27.5)	6(11.8)		
	25-40	78	11(14.1)	18(23.1)	35(44.9)	14(17.9)		
	41-60	41	4(9.8)	13(31.7)	18(43.9)	6(14.6)		
	>60	2	0(0.0)	1(50.0)	1(50.0)	0(0.0)		
Eruption, n (%)	No	19	5(26.3)	12(63.2)	1(5.3)	1(5.3)	16.222	0.001
	Yes	156	21(13.5)	43(27.6)	67(42.9)	25(16.0)		
Horizontal, n (%)	A	95	8(8.4)	21(22.1)	51(53.7)	15(15.8)	30.078	<0.001
	B	56	14(25.0)	19(33.9)	15(26.8)	8(14.3)		
	C	24	4(16.7)	15(62.5)	2(8.3)	3(12.5)		
Vertical, n (%)	I	95	11(11.6)	18(18.9)	50(52.6)	16(16.8)	30.839	<0.001
	II	53	12(22.6)	19(35.8)	14(26.4)	8(15.1)		
	III	27	3(11.5)	18(32.7)	4(5.9)	2(7.7)		
Degree of impaction, n (%)	Shallowness obstruction	148	23(15.5)	37(25.0)	64(43.2)	24(16.2)	18.857	<0.001
	Deep obstruction	27	3(11.1)	18(66.7)	4(14.8)	2(7.4)		
Number of roots, n (%)	Absent	21	8(38.1)	11(52.4)	1(4.8)	1(4.8)	30.085	<0.001
	Single root	29	3(10.3)	15(51.7)	8(27.6)	3(10.3)		
	Follow more	125	15(12.0)	29(23.2)	59(47.2)	22(17.6)		
Angle of occlusal plane and M3 longitudinal axis, n (%)	Distoangular(>100°)	5	1(20.0)	2(40.0)	2(40.0)	0(0.0)	6.780	0.674
	Vertical(81-100°)	57	7(12.3)	13(22.8)	29(50.9)	8(14.0)		
	Mesioangular (21-80°)	92	15(16.3)	33(35.9)	30(32.6)	14(15.2)		
	Horizontal (≤20°)	21	3(14.3)	7(33.3)	7(33.3)	4(19.0)		
Impacted, n (%)	No	84	6(7.1)	18(21.4)	47(56.0)	13(15.5)	23.801	<0.001
	Yes	91	20(22.0)	37(40.7)	21(23.1)	13(14.3)		
Fracture type, n (%)	Angle Fracture Group	70	16(22.9)	25(35.7)	18(25.7)	11(15.7)	20.270	0.002
	Condylar Fracture Group	77	10(13.0)	25(32.5)	35(45.5)	7(9.1)		
	Mandibular chin fracture group	28	0(0.0)	5(17.9)	15(53.6)	8(28.6)		

**Table 2:** Non-fracture group B/A.

Characteristics		Total n	<0.5	0.5-0.6	0.61-0.7	>0.7	χ2	P
Gender, n (%)	Male	39	5(12.8)	7(17.9)	17(43.6)	10(25.6)	3.160	0.368
	Female	28	4(14.3)	10(35.7)	8(28.6)	6(21.4)		
Age, yr, n (%)	<14	4	3(75.0)	1(25.0)	0(0.0)	0(0.0)	20.279	0.056
	14-24	18	2(11.1)	6(33.3)	6(33.3)	4(22.2)		
	25-40	20	1(5.0)	6(30.0)	7(35.0)	6(30.0)		
	41-60	21	3(14.3)	3(14.3)	11(52.4)	4(19.0)		
	>60	4	0(0.0)	1(25.0)	1(25.0)	2(50.0)		
Eruption, n (%)	No	7	5(71.4)	1(14.3)	1(14.3)	0(0.0)	22.929	<0.001
	Yes	60	4(6.7)	16(26.7)	24(40.0)	16(26.7)		
Horizontal,n (%) o	A	36	2(5.6)	11(30.6)	15(41.7)	8(22.2)	8.876	0.175
	B	27	5(18.5)	5(18.5)	9(33.3)	8(29.6)		
	C	4	2(50.0)	1(25.0)	1(25.0)	0(0.0)		
Vertical, n (%)	I	35	2(5.7)	10(28.6)	14(40.0)	9(25.7)	14.506	0.022
	II	21	2(9.5)	4(19.0)	8(38.1)	7(33.3)		
	III	11	5(45.5)	3(27.3)	3(27.3)	0(0.0)		
Degree of impaction, n (%)	Shallowness obstruction(IA,IB,IIA, IIB,IIIA)	54	2(3.7)	14(25.9)	22(40.7)	16(29.6)	24.373	<0.001
	Deep obstruction (IC,IIC,IIIB,IIIC)	13	7(53.8)	3(23.1)*	3(23.1)*	0(0.0)*		
Number of roots ,n (%)	Absent	7	4(57.1)	3(42.9)	0(0.0)	0(0.0)	18.814	0.004
	Single root	5	0(0.0)	2(40.0)	1(20.0)	2(40.0)		
	Follow more	55	5(9.1)	12(21.8)	24(43.6)	14(25.5)		
Angle of occlusal plane and M3 longitudinal axis,n (%)	Vertical(81-100°)	19	3(15.8)	5(26.3)	9(47.4)	2(10.5)	12.138	0.057
	Mesioangular (21-80°)	27	6(22.2)	9(33.3)	6(22.2)	6(22.2)		
	Horizontal (≤20°)	21	0(0.0)	3(14.3)	10(47.6)	8(38.1)		
Impacted,n (%)	No	30	1(3.3)	10(33.3)	12(40.0)	7(23.3)	5.594	0.138
	Yes	37	8(21.6)	7(18.9)	13(35.1)	9(24.3)		

Note: * Compared with group B/A < 0.5, P < 0.05.

**Table 3:** General data of iatrogenic mandibular fracture.

Patients	Gender	Age	Postoperative Day	B/A	Dental position	Classification
A	M	27	20	0.38	38	IIC
B	M	32	22	0.35	38	IIC
C	M	36	25	0.33	38	IIC
D	M	52	15	0.22	38	IIC
E*	F	40	0	0.47	38	IIB
F	F	53	0	0.12	48	IIC
G	F	36	0	0.25	48	IIIC
H*	F	37	0	0.37	37	not classified
I	F	20	10	0.19	48	IIC
J	F	30	0	0.32	48	N
K	M	44	28	0.29	48	IIB
L	F	59	14	0.27	48	IIC
M	F	63	28	0.23	38	IIB
N	F	34	0	0.19	48	N
O	F	37	0	0.17	38	IB
P	F	26	0	0.35	48	IIB
Q	F	18	0	0.18	38	IIIC
R	Å®	35	0	0.30	48	IIC
S	Å®	33	10	0.39	48	IIC
T*	Å®	25	0	0.28	48	IIIA

Note: * case E mandibular ramus fracture, * case H body fracture, * case T mandibular angle osteotomy and tooth extraction leading to fracture.

**Table 4:** Comparison of four groups of B/A values.

Groups	BA
Normal non-fracture group	0.62±0.10*
Traumatic fracture group	0.61±0.10*
Iatrogenic fracture group	0.28±0.08
Impacted tooth extraction group	0.62±0.09*
*F*	72.41
*P*	<0.001

Note: * Compared with the iatrogenic fracture group, *P* < 0.05.

## Data Availability

The datasets used and/or analysed during the current study available from the Correspondence on reasonable request. We declared that materials described in the manuscript, including all relevant raw data, will be freely available to any scientist wishing to use them for non-commercial purposes, without breaching participant confidentiality.
